# Development of a prediction model integrating PD-1 and ICOS for early differential diagnosis between autoimmune and viral encephalitis

**DOI:** 10.3389/fimmu.2025.1550963

**Published:** 2025-04-25

**Authors:** Kaiyue Xu, Jinjing Jia, Yinghui Duan, Shuying Chen, Xinyi Xiao, Feng Zhu, Xin Wang, Yanzheng Gu, Jingluan Tian, Qun Xue

**Affiliations:** ^1^ Department of Neurology, The First Affiliated Hospital of Soochow University, Suzhou, China; ^2^ Jiangsu Institute of Clinical Immunology, Jiangsu Key Laboratory of Clinical Immunology, The First Affiliated Hospital of Soochow University, Suzhou, China

**Keywords:** co-stimulatory molecules, autoimmune encephalitis, viral encephalitis, differential diagnosis, predictive model

## Abstract

**Background:**

Early diagnosis and treatment for encephalitis are crucial for improving patient outcomes and reducing the economic burden, especially given the overlapping symptoms and low specificity of auxiliary diagnostic tests between viral encephalitis (VE) and autoimmune encephalitis (AE). Since these two conditions require different treatment approaches, an early differential diagnosis between AE and VE is a critical challenge.

**Methods:**

This study enrolled a cohort of 75 patients (38 with VE and 37 with AE) between September 2022 and July 2024. The demographic data, clinical characteristics, and laboratory test results were collected. The expression levels of co-stimulatory molecules were detected by flow cytometry and enzyme-linked immunosorbent assay within 7 days for viral encephalitis and 90 days for autoimmune encephalitis in the early phase of the disease. Differential analysis, logistic regression analysis, and least absolute shrinkage and selection operator regression were employed for model construction. Finally, a nomogram and a receiver operating characteristic (ROC) curve were developed to visualize the model and evaluate its predictive accuracy.

**Results:**

Upon analyzing the collected data, a model for the early differential diagnosis between AE and VE was eventually established. This comprehensive model incorporated 10 variables: serum creatinine and chloride levels, the percentage of peripheral blood CD4^+^ICOS^+^ and CD19^+^PD-L1^+^, plasma soluble inducible costimulatory ligand (sICOSL), cerebrospinal fluid (CSF) glucose content, and the presence of fever, nausea, vomiting, headaches, and cognitive impairment. Patients with creatinine <60.75 (μmol/L), chloride <106.25 (mmol/L), CD4^+^ICOS^+^ ≥11.2%, CD19^+^PD-L1^+^ ≥12.35%, plasma sICOSL≥286.37 ng/mL, CSF sugar content ≥3.775 (mmol/L), and those with cognitive impairment are more likely to be diagnosed with AE. The area under the curve (AUC)-ROC of our model was 0.942 [95% confidence interval (CI): 0.887–0.997], with a sensitivity of 0.844 and a specificity of 0.971, indicating strong diagnostic performance.

**Conclusion:**

This diagnostic model offers a convenient tool for distinguishing AE from VE in the early phase, facilitating early diagnosis and treatment, improving patient prognosis, and reducing financial burdens.

## 1 Introduction

Encephalitis is an inflammatory disorder of brain tissue that may present with either diffuse or focal neuropsychological dysfunction. It is characterized by high mortality and morbidity rates, as well as severe neurological symptoms. It affects individuals across all age groups and poses a significant burden on patients, families, and society. The primary etiologies of encephalitis include infectious and non-infectious origins, with viral infections being the most common among infectious cases. Autoimmune disorders represent the third most prevalent cause of encephalitis, following infections and acute disseminated encephalomyelitis (ADEM) (1). The multiple types of infectious and autoimmune causes highlight the significant variability in the clinical presentation of encephalitis. In clinical practice, differentiating infectious encephalitis (IE) from autoimmune encephalitis (AE) is crucial due to their distinct treatment strategies and prognoses. IE is caused by various pathogens, including bacteria, fungi, and viruses. Cerebrospinal fluid (CSF) profiles in bacterial or fungal infections typically differ from those in viral infections and AE, making them easier to distinguish from AE. Bacterial infections typically show an increase in lactate concentration in CSF, a lower ratio of CSF to serum glucose (2), and a significant increase in white blood cells. Both viral infections and AE are associated with a mild increase in white blood cells (predominantly lymphocytes) and elevated protein levels in the CSF (3).

Viral encephalitis (VE) is an acute infectious disease of the central nervous system caused by a wide range of viral infections, characterized by a sudden onset of psychiatric and behavioral abnormalities, decreased consciousness level, and seizures, which are sometimes irreversible (4). The severity of viral encephalitis is determined by the interaction between the host immune response and the invading virus. Thus, prompt diagnosis and treatment are imperative. The most frequent cause of viral encephalitis is herpes simplex virus, while other sources include varicella virus (5). Herpes simplex virus encephalitis (HSE) has a high mortality and disability rate. The death rate of HSE patients who have not received antiviral treatment is approximately 70%, and most patients are left with permanent neurological sequelae (6). Currently, acyclovir is the preferred drug for treating HSE (6).

AE is a group of inflammatory brain disorders mediated by abnormal immune responses targeting neuronal antigens and characterized by cognitive impairment, seizures, and psychiatric and behavioral disturbances (7). Over the past two decades, autoimmune encephalitis has gained increasing recognition and has become an important differential diagnosis for patients presenting with rapidly progressive cognitive impairment, psychiatric and behavioral abnormalities, focal neurological symptoms, and seizures (8). AE is associated with various auto-antibodies targeting neuronal surface antigens or intracellular antigens. The neuronal surface antibodies include anti-N-methyl-D-aspartate receptor (NMDAR), anti-leucine-rich glioma-inactivated 1(LGI1), anti- gamma aminobutyric acid receptor (GABABR), and anti-dopamine D2 receptor (D2R) antibodies; intracellular antibodies include anti-Hu (anti-neuronal nuclear antibody type 1) antibody, anti-Ma2/Ta antibody, and anti-glutamic acid decarboxylase (GAD) antibodies. Some types of AE, such as anti-Hu encephalitis and anti-Ma2 encephalitis, are categorized as neurological paraneoplastic syndrome and are linked to tumors,. Among these, anti-NMDAR encephalitis accounts for 10%–20% of all cerebral infections and is the most prevalent subtype (9). Previous studies showed that AE commonly exhibits with prodromal symptoms, such as headache, fever, and diarrhea, which may suggest an associated infectious episode. The connection between AE and preceding infection has been extensively explored, particularly in cases involving anti-NMDAR encephalitis secondary to HSE with typical bimodal encephalitis.

AE and VE share similar clinical manifestations and laboratory findings (10), while diagnostic tools such as electroencephalogram (EEG) and magnetic resonance imaging (MRI) lack adequate specificity, highlighting the importance of differentiation (11). Furthermore, the management of AE differs from that of VE. AE is primarily treated with plasma exchange and immunosuppressive therapy such as high-dose steroids (12, 13), whereas antiviral therapy, such as low-dose steroids, is the primary treatment for VE (14),. Several research studies have demonstrated that early proper intervention can significantly improve the prognosis of patients with encephalitis (12). Initiating immune therapy at disease onset has been associated with a reduced likelihood of relapse (15, 16), and delayed treatment is a risk factor for relapse. The prognosis and recurrence of AE are closely related to early treatment.

The early differential diagnosis between AE and VE is pivotal for ensuring timely and appropriate treatment. Currently, the diagnosis of AE requires antibody testing and differential diagnosis with other diseases, and the diagnosis of VE relies on pathogen metagenomic next-generation sequencing (mNGS) or other pathogen detection methods (17). In fact, antibody testing plays a pivotal role in the early diagnosis of AE, but it exhibits a low positive rate (9), subjective interpretation, prolonged detection time, and substantial cost. The current diagnostic criteria for AE places significant emphasis on antibody testing and the effectiveness of immunotherapy, potentially leading to delays in timely diagnosis. As for mNGS, its positive rate is low, and the cost is high (17). Consequently, further investigation is warranted to devise early differential diagnostic approaches between AE and VE to facilitate prompt intervention.

In this study, we collected the demographic data, clinical features, laboratory test results, and flow cytometry results of 38 patients with VE and 37 patients with AE in the early stage to comprehensively investigate the distinctions between early VE and AE, with the aim of developing a clinical prediction model for differential diagnosis to supply rational approaches to the selection of therapeutics.

## 2 Materials and methods

### 2.1 Study design

**Figure 1:**
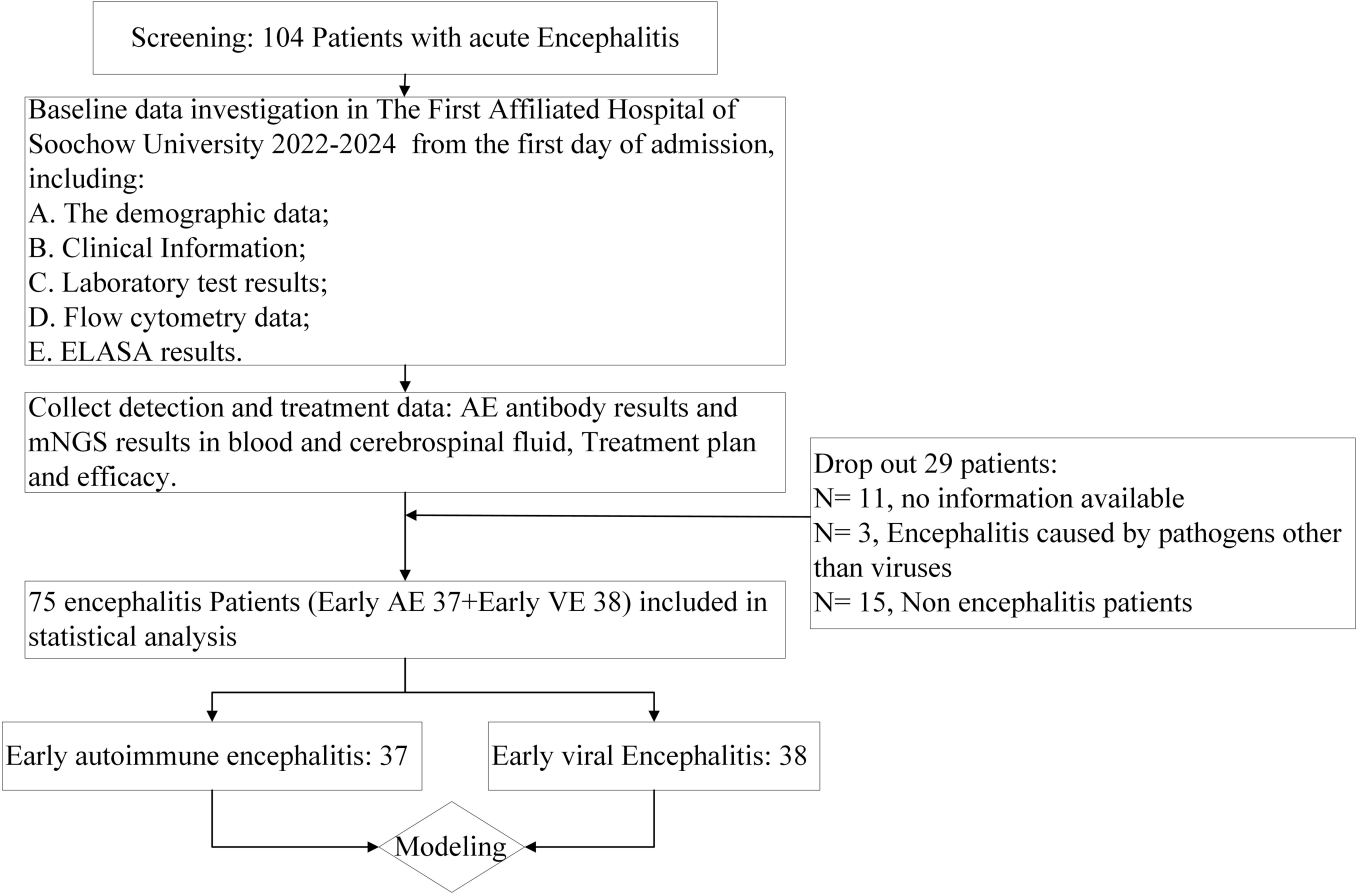
Flow chart. ELISA, enzyme-linked immunosorbent assay; mNGS, metagenomic next-generation sequencing; AE, autoimmune encephalitis; VE, viral encephalitis.

This study was conducted from September 2022 to July 2024 at The First Affiliated Hospital of Soochow University and involved hospitalized patients with suspected encephalitis. Patients admitted with neurological disorders were selected based on the diagnostic criteria for encephalitis proposed by Arun et al. in 2019 (18). This study was approved by the Ethics Committee of the First Affiliated Hospital of Soochow University, and all participants provided informed consent. The demographic data, clinical characteristics, laboratory test results, and flow cytometry analysis results from the patients on their first day of admission were collected. In accordance with the predefined inclusion and exclusion criteria, patients who were clinically diagnosed with AE and VE were enrolled in this study, thereby establishing the respective AE and VE research cohorts for comparative analysis. Autoimmune encephalitis antibody testing and pathogen mNGS results were obtained from accredited institutions (Nanjing Simcere Medical Laboratory Science, Hangzhou Oumeng Weiyi Medical Laboratory, HUGO BIOTECH), and the treatment outcomes were collected. The enrolled patients were subjected to statistical analysis, which facilitated the development of a clinical diagnostic model (**Figure 1**).

### 2.2 Patients with AE or VE

All AE patients met the diagnostic criteria outlined in the 2022 Chinese Expert Consensus on the Diagnosis and Management of Autoimmune Encephalitis (19) and the criteria for autoimmune encephalitis established by Graus et al. in 2016 (20). Patients with possible AE were enrolled. The inclusion criteria for the AE group required patients to be within 3 months of symptom onset and to fulfill the following four criteria: (1) presence of at least one of the following symptoms: psychiatric and behavioral disturbances, cognitive impairment, seizure disorders, consciousness disorders, and motor disorders; (2) fulfillment of at least one of the following: elevated white blood cell count in cerebrospinal fluid (CSF) or presence of oligoclonal bands, electroencephalographic (EEG) abnormalities characteristics of encephalitis, neuroimaging findings consistent with encephalitis, or association with tumors; (3) detection of positive CSF and serum antibodies against neuronal synaptic or cell surface antigens; and (4) no prior immunotherapy before admission. The exclusion criteria for AE are as follows: (1) patients who have undergone immunotherapy or (2) patients with AE who also have infections, such as viral or bacterial infections.

The diagnostic criteria for viral encephalitis were based on guidelines established by Hongzhi et al. in 2022 (21) and Tyler et al. in 2018 (22). The inclusion criteria required patients to meet all of the following: (1) clinical manifestations, including headache, new seizure disorders, consciousness disorders, and persistent mental and behavioral disorders, lasting for at least 24 hours; (2) evidence of infection, including fever (body temperature ≥ 38°C) before or within 72 hours of symptom onset, CSF inflammation, neuroimaging abnormalities in the brain parenchyma, or characteristic EEG abnormalities indicative of encephalitis; (3) response to antiviral therapy or positive identification of viral nucleic acids or antibodies in the CSF or serum; and (4) no prior treatment before admission.

Exclusion criteria for VE and AE included: (1) encephalitis resulting from non-viral etiologies, including bacterial and fungal agents; (2) incomplete clinical data; or (3) patients who have been excluded as potential encephalitis cases. To minimize potential confounding effects, patients with diabetes were deliberately excluded due to the influence of blood glucose levels on CSF glucose concentration.

### 2.3 Clinical symptoms and laboratory test

Demographic data, clinical characteristics, and laboratory test results were collected on their first day of admission (AE patients: onset within 3 months; VE patients: onset within 7 days). Treatment outcomes were followed up.

Demographic data included name, gender, and age of onset. Clinical characteristics were assessed based on a broad range of early-phase symptoms, including fever, headache, upper respiratory symptoms (cough, phlegm, nasal congestion, and sneezing), nausea, vomiting, consciousness disorders, psychiatric and behavioral disturbances, seizure disorders, involuntary movements, cognitive impairment, ataxia, and sleep disorders.

The laboratory test results were categorized into peripheral blood and CSF analyses. The blood analysis included analysis of blood cells, a full-set immunity biochemical panel (including lymphocyte subpopulations and humoral immunity markers), and the neutrophil-to-lymphocyte ratio (NLR). The CSF examination included the CSF routine test, biochemical analysis, immunofixation electrophoresis, and oligoclonal band detection.

### 2.4 Flow cytometry

The peripheral blood samples were collected into Ethylenediaminetetraacetic acid (EDTA) anticoagulation tubes upon admission, and membrane co-stimulatory molecules were promptly detected by flow cytometry. The sequential procedures are briefly as followed: 50µL peripheral blood was incubated with the following fluorescently labeled monoclonal antibodies for 30min in dark: PE/Cyanine 7 anti-human CD4 (300512), FITC anti-human/mouse/ral CD278 (ICOS) (313506), FITC anti-human CD19 (392508), PerCP/Cyanine 5.5 anti-human CD279 (PD-1) (329914), PE-anti-human CD185 (CXCR5) (356904), PE/Cyanine 7 anti-human CD14 (367112), APC anti-human CD275 (B7H2, ICOSL) (309408), and PerCP/Cyanine5.5 anti-human CD274(B7-H1, PD-L1) (329738) (all antibodies were purchased from BioLegend, San Diego, CA, USA). Subsequently, 200 µL of red blood cell lysis buffer (Beckman Coulter, USA) was added to the blood sample, followed by incubation in a 37°C constant temperature water bath for 15 minutes until the sample became clear and free of precipitate. After incubation, the lysis reaction was terminated by adding 1 mL of phosphate-buffered saline (PBS). The sample was then centrifuged at 1,200 rpm for 5 minutes, and the resulting cell pellet was resuspended in PBS for flow cytometric analysis (Beckman Coulter, USA). FlowJo version 10 software was used to analyze the primary flow cytometry data.

### 2.5 Enzyme-linked immunosorbent assays

Plasma and CSF samples stored at -80°C were thawed overnight at 4°C. Repeated freezing and thawing should be avoided. Plasma and CSF supernatants were collected for enzyme-linked immunosorbent assays (ELISAs). A human T-cell inducible costimulator (ICOS) ELISA kit (Kanglang Biotechnology, China) was used to detect the concentration of plasma soluble ICOS (sICOS). Plasma levels of soluble T-cell inducible costimulator ligand (sICOSL), soluble programmed death-1 (sPD-1), and soluble programmed death-ligand 1 (sPD-L1) were measured with human ICOSL, PD-1, and PD-L1 ELISA kits (BSABIO, China). The human cluster of differentiation 14 (CD14) ELISA kit (BSABIO, China) was used to quantify plasma soluble CD14 (sCD14). Each ELISA was performed according to the manufacturer’s instructions. This study required precise preparation of standard solutions, wash buffers, and stop solutions. When adding samples, blank wells, standard wells, and sample wells were set up on the enzyme-linked immunosorbent assay plate. In the standard wells, 50 μL of the standard solution was precisely added. In the sample wells, 40 μL of sample diluent was added first, followed by 10 μL of sample, achieving a final dilution factor of 5. The plate was sealed and incubated at 37°C for 30 minutes. The liquid was discarded, and each well was filled with wash buffer, allowed to stand for 30 seconds, and then aspirated. The washing procedure was repeated five times. Following the final wash, 50 μL of enzyme conjugate was added to each well, except for blank wells. The incubation and washing steps were repeated as previously described. Next, 50 μL of chromogen substrate A was added, followed by 50 μL of chromogen substrate B, and the plate was incubated at 37°C in the dark for 10 minutes to allow color development. Finally, 50 μL of stop solution was added to each well to terminate the reaction. Absorbance was measured using a microplate reader (Bio-Rad Laboratories, Inc., USA).

### 2.6 Statistical analyses

SPSS version 26.0 and GraphPad Prism 10.0 were used for statistical analyses, while flow cytometry data were analyzed using FlowJo version 10. The entire dataset was used to construct the predictive model, with no variable containing more than 5% missing data. Quantitative data were tested for normality, and variables following a normal distribution were expressed as mean ± standard deviation (x ─ ± s). For non-normally distribution data, results were presented as the median (M) with interquartile range (IQR: P25, P75). The count data are represented by the frequency (n) and percentage composition (%). Comparative analysis of clinical data required appropriate statistical tests. Specifically, independent sample t-tests were employed to analyze normally distributed quantitative data, while Mann-Whitney U non-parametric tests were used for non-normally distributed quantitative data. Additionally, Chi-square tests were used to analyze categorical variables. Afterward, the variables in the two groups underwent single-factor logistic regression analysis, and only the variables with a significance level of p < 0.05 were further subjected to least absolute shrinkage and selection operator (LASSO) regression analysis. Subsequently, a LASSO multiple linear regression model was constructed, and the standardized equation of this model was obtained. A nomogram was constructed for model visualization, enabling the prediction of individual patient disease probabilities. Finally, a receiver operating characteristic (ROC) curve was generated to assess the model’s predictive accuracy. This study lacked an independent validation cohort to verify the model.

## 3 Results

### 3.1 Demographics and clinical manifestation

A total of 104 patients were screened, of whom 29 were excluded for not meeting the diagnostic criteria for AE or VE or due to insufficient clinical information. Ultimately, 75 patients were included in the study, comprising 38 patients with VE and 37 with AE. The distribution of antibody types among the patients with AE is presented as follows: NMDAR antibody, four cases; LGI1 antibody, three cases; GABABR antibody, three cases; GAD-65 antibody, two cases; anti-myelin oligodendrocyte glycoprotein (MOG) antibody, four cases; anti-Hu antibody, one case; and anti-Ri antibody, one case. The remaining AE patients tested positive using the tissue-based assay (TBA), but their specific antibody type was not identified.

AE and VE were observed in both men and women. The median age of onset was 48 years in the AE group and 40 years in the VE group. There were no statistically significant differences in gender or age at onset between the two groups (P > 0.05) (**Table 1**).

**Table 1:** Comparison of the demographic data and clinical features between patients with AE and VE.

	AE (n=37)	VE (n=38)	Z/X^2^	P-value
Demographic data				
Age of onset	48 (25,62)	40 (34,50)	-1.13	0.257
Gender (Female)	17 (45%)	16 (42%)	0.054	0.817
Clinical data				
Fever	12 (32%)	29 (76%)	15.306	**<0.001**
Tmax (°C)^a^	39.3 (39.3,39.3)	39.3 (39,39.4)	-1	0.316
Nausea and vomiting	6 (16%)	17 (45%)	7.544	**0.006**
Upper respiratory symptoms	7 (18%)	15 (39%)	4.094	**0.043**
Headache	12 (32%)	23 (61%)	6.408	**0.011**
Consciousness disorders	16 (42%)	16 (42%)	0	1
Mental and behavioral disorders	19 (50%)	8 (21%)	6.951	**0.008**
Seizure disorders	9 (24%)	7 (18%)	0.317	0.574
Involuntary movements	9 (24%)	5 (13%)	1.401	0.237
Cognitive impairment	13 (34%)	5 (13%)	4.659	**0.031**
Ataxia	17 (45%)	12 (32%)	2.645	0.267
Sleep disorders	2 (5%)	7 (18%)	3.151	0.076

Values are presented as number (%) or median (M), 25% interquartile range (Q1), and 75% interquartile range (Q3). Tmax, body temperature maximum, the maximum temperature of the patient since the disease onset; AE, autoimmune encephalitis; VE, viral encephalitis.

Boldface values (all <0.05) indicate statistically significant differences in the corresponding variables between autoimmune encephalitis and viral encephalitis groups.

The early manifestations of VE were similar to those of AE. Early AE was predominantly characterized by psychiatric and behavioral disturbances, as well as cognitive impairment. In contrast, early VE was marked by fever, nausea and vomiting, upper respiratory symptoms, and headache. Statistically significant differences were found between the two groups (P < 0.05). However, there were no statistically significant differences between the two groups in terms of consciousness disorders, epilepsy, urinary and fecal incontinence, involuntary movements, ataxia, or sleep disturbances (P > 0.05) (**Table 1**).

### 3.2 Laboratory evaluations

The levels of peripheral blood lactate dehydrogenase (LDH) and calcium were significantly elevated in the early AE group compared to the early VE group (P < 0.05). In contrast, chloride and creatinine levels were significantly higher in the early VE group than in the early AE group (P < 0.05). These four parameters demonstrated statistically significant differences between the early AE and VE groups. However, no significant differences were observed in other blood biochemical indicators between the two groups (**Table 2**).

**Table 2:** Comparison of chemistry panel results between autoimmune encephalitis and viral encephalitis.

	AE(n=37)	VE(n=38)	t/Z	P-value
TBil (umol/L)^a^	12.8 (7.45, 16.1)	9.7 (6.3, 15.2)	-1.17	0.241
DBil (umol/L)^b^	4 (2.95, 5.95)	3.4 (2.2, 5.2)	-1.11	0.268
IBIL (umol/L)^c^	8.08 (5.05, 10.3)	6.1 (3.95, 9.7)	-1.61	0.107
Glucose (mmol/L)	5.09 (4.51, 6.31)	4.96 (4.56, 5.89)	-0.94	0.35
ALT (U/L)^d^	18.4 (11.75, 33.56)	24.3 (12.5, 33.1)	-0.34	0.737
AST (U/L)^e^	17 (12.9, 25.1)	16.6 (12.55, 28.65)	-0.07	0.944
GGT (U/L)^f^	24.4 (15.3, 40.2)	23.6 (15, 43.95)	-0.08	0.939
Albumin (g/L)	40.1 (36.25, 43.75)	38.6 (36.35, 42.2)	-0.76	0.449
Globulin (g/L)	26.69 (22.45, 29.85)	25.1 (23.9, 27.8)	-0.43	0.665
Urea (mmol/L)	5.3 (4, 6.45)	4.6 (3.65, 5.8)	-1.51	0.13
Cystatin C (mg/L)	0.9 (0.84, 1.02)	0.83 (0.76, 0.97)	-1.62	0.105
TG (mmol/L)^g^	1.17 (0.89, 1.49)	1.13 (0.75, 1.37)	-0.81	0.417
LDL (mmol/L)^h^	2.71 (2.01, 3.04)	2.81 (2.09, 3.27)	-0.82	0.414
Potassium (mmol/L)	3.8 (3.61, 4)	3.94 (3.71, 4.05)	-1.37	0.171
Sodium (mmol/L)	139.4 (136.55, 141.45)	139.7 (137.75, 142.7)	-0.96	0.339
Chloride (mmol/L)	102.5 (100.5, 104.45)	103.9 (102.05, 107.25)	-2.2	**0.028**
LDH (U/L)^i^	187 (151.3, 226.45)	160.7 (139.75, 184.45)	-2.11	**0.035**
CK (U/L)^j^	63.7 (38.6, 183.18)	59.4 (50.75, 185.5)	-0.26	0.791
α-HBDH (U/L)^k^	135.7 (117.25, 176.4)	125.2 (105.85, 155.55)	-1.25	0.21
CRP (mg/L)^l^	3.06 (0.58, 7.58)	2.56 (1.03, 10.13)	-0.55	0.584
TC (mmol/L)^m^	4.48 ± 1.06	4.24 ± 0.93	1.01	0.32
ALP (U/L)^n^	73.13 ± 25.84	65.11 ± 20.68	1.47	0.15
Total protein (g/L)	66.48 ± 12.31	64.28 ± 6.5	0.96	0.34
Prealbumin (g/L)	229.83 ± 69.54	232 ± 67.07	-0.14	0.89
Creatinine (umol/L)	56.49 ± 14.33	63.64 ± 16.42	-1.99	**0.05**
Uric acid (umol/L)	225.98 ± 96.21	228.21 ± 95.7	-0.1	0.92
HDL (mmol/L)°	1.15 ± 0.45	1 ± 0.23	1.74	0.09
Calcium (mmol/L)	2.3 ± 0.13	2.22 ± 0.15	2.33	**0.02**
Phosphorus ion (mmol/L)	1.23 ± 0.28	1.12 ± 0.26	1.79	0.08
Iron (umol/L)	14.05 ± 6	12.83 ± 5.44	0.92	0.36

Values are presented as mean ± Standard Deviation or median (M), 25% interquartile range (Q1), and 75% interquartile range (Q3). TBil, total bilirubin; DBil, direct bilirubin; IBIL, indirect bilirubin; ALT, alanine aminotransferase; AST, aspartate aminotransferase; GGT, gamma-glutamyl transferase; TG, triglycerides; LDL, low-density lipoprotein; LDH, lactate dehydrogenase; CK, creatine kinase; α-HBDH, α-hydroxybutyric dehydrogenase; CRP, C-reactive protein; TC, total cholesterol; ALP, alkaline phosphatase; HDL, high-density lipoprotein. AE, autoimmune encephalitis; VE, viral encephalitis.

Boldface values (all <0.05) indicate statistically significant differences in the corresponding variables between autoimmune encephalitis and viral encephalitis groups.

In terms of CSF examination, the cerebrospinal fluid pressure and white blood cell count were significantly lower in the early AE group compared to the early VE group, while the glucose content in the CSF was significantly higher in the early AE group than in the early VE group. These differences between the two groups exhibited statistical significance (P < 0.05). Additionally, while serum immunoglobulin G (IgG) levels were significantly higher in the AE group compared to the VE group (P < 0.05), no statistically significant difference was observed in CSF IgG levels between the two groups. Furthermore, other parameters measured in CSF samples showed no significant differences between the two groups (P > 0.05) (**Table 3**).

**Table 3:** Comparison of CSF examination results between autoimmune encephalitis and viral encephalitis.

	AE (n=37)	VE (n=38)	Z/X^2^	P-value
CSF pressure (mmH2O)	145 (120, 157)	157 (150, 180)	-2.82	**0.005**
CSF white blood cell count (10^6/L)	14 (1.5, 47.4)	42.7 (3, 78.25)	-2	**0.045**
CSF red blood cell count (10^6/L)	0 (0, 0)	0 (0, 0)	-0.7	0.482
CSF MNC percentage (%) ^b^	78.6 (50, 98.4)	96.25 (59.05, 98.7)	-0.67	0.506
CSF PMN percentage (%)^c^	14.3 (0, 35.95)	2.95 (0.98, 23.58)	-0.49	0.627
HF-BF% (/100 WBC) ^d^	0.96 (0.96, 0.96)	0.96 (0.3, 0.96)	-1.03	0.301
CSF chlorides (mmol/L)	121.9 (120.1, 124.4)	121.74 (119.48, 124.1)	-0.75	0.451
CSF protein (g/L)	0.53 (0.27, 0.63)	0.42 (0.28, 0.97)	-0.1	0.917
CSF glucose (mmol/L)	3.78 (3.36, 4.41)	3.57 (3.12, 3.8)	-1.97	**0.048**
CSF ADA (U/L) ^e^	0.6 (0.2, 1.07)	0.75 (0.18, 1.6)	-0.47	0.635
CSF LDH (U/L) ^f^	22 (16.5, 29.85)	22.5 (16.5, 29)	-0.17	0.865
CSF albumin (mg/L)	416 (169, 444.66)	392.83 (154.5, 678.75)	-0.26	0.795
Serum albumin (g/L)	38.45 (34.13, 40.68)	36.2 (34.4, 40.68)	-0.49	0.627
CSF QIgG^g^	6.72 (2.52, 8.12)	7.47 (2.05, 10.29)	-0.13	0.896
CSF IgG index^h^	0.61 (0.49, 0.73)	0.59 (0.51, 0.72)	-0.59	0.556
CSF Q(A1b) ^i^	12.11 (4.48, 12.45)	10.84 (4.23, 17.6)	-0.33	0.743
CSF 24h intrathecal synthesis rate (mg/24h)	6.27 (-0.37, 11.61)	5.01 (-1.18, 11.61)	-0.07	0.946
CSF IgG (mg/L) ^j^	72.5 (25.25, 88.69)	86.15 (26, 104.78)	-0.27	0.786
Serum IgG (g/L)	11.35 (8.78, 12.39)	11.05 (9.29, 12.39)	-0.29	0.773
CSF Pandy’s test	15 (39%)	13 (34%)	0.226	0.634
CSF OCBs (+)^k^	3 (8%)	2 (5%)	0.187	0.666
Serum OCBs (+)	4 (11%)	0 (0%)	4.229	**0.04**
CSF pathology (lymphocytes)	11 (29%)	12 (32%)	0.062	0.803
CSF pathology (atypical Cells)	8 (21%)	17 (45%)	4.828	**0.028**

Values are presented as number (%) or median (M), 25% interquartile Range (Q1), and 75% interquartile range (Q3). CSF, cerebrospinal fluid; CSF MNC percentage, CSF mononuclear cell percentage; CSF PMN percentage, CSF polymorphonuclear cell percentage; HF-BF%, high fluorescence intensity cells percentage; CSF ADA, CSF adenosine deaminase; CSF LDH, CSF lactate dehydrogenase; CSF QIgG, CSF/serum IgG gradient; CSF IgG index, CSF immunoglobulin G index; CSF Q(A1b), CSF albumin ratio; CSF IgG, CSF immunoglobulin G; CSF OCBs, CSF oligoclonal bands. AE, autoimmune encephalitis; VE, viral encephalitis.

In the full-set immunity analysis, the CD4^+^/CD8^+^ ratio and B factor levels were significantly higher in the early VE group compared to the early AE group (P < 0.05), indicating statistically significant differences in these three indicators between the two groups. There were no statistically significant differences observed between the two groups in terms of CD3^+^ (%), CD3^+^CD4^+^ (%), CD3^-^CD19^+^(%), immunoglobulin A, complement 4, CD3^+^CD8^+^ (%), CD3^-^CD (16 + 56) ^+(^%), immunoglobulin G, immunoglobulin M, kappa-light chain, and lambda-light chain levels (P > 0.05) (**Table 4**). The blood analysis included monocyte counts, neutrophil counts and percentages, hemoglobin levels, platelet counts, red blood cell counts, white blood cell counts, lymphocyte counts and percentages, monocyte percentages, and the NLR. No significant differences were observed in these parameters between the early AE and VE groups (P > 0.05) (**Supplementary Table 1**).

**Table 4:** Comparison of full-set immunity biochemical panel results between autoimmune encephalitis and viral encephalitis.

	AE (n=37)	VE(n=38)	t/Z	P-value
CD3^+^ (%)	68.64 ± 10.33	66.87 ± 12.84	0.47	0.64
CD3^+^CD4^+^ (%)	35.11 ± 10.78	36 ± 9.98	-0.25	0.8
CD3^-^cD19^+^ (%)	17.6 ± 8.4	18.27 ± 9.08	-0.23	0.82
Immunoglobulin A(g/L)	2.15 ± 1.04	2.16 ± 0.7	-0.06	0.95
Complement 3(g/L)	0.88 ± 0.29	0.95 ± 0.21	-0.75	0.46
CD3^+^CD8^+^ (%)	28.24 (26.24, 32.05)	21.72 (19.76, 33.5)	-1.61	0.11
CD4^+^/CD8^+^	1.14 (0.91, 1.55)	1.72 (1.16, 1.97)	-2.02	**0.04**
CD3^-^CD (16 + 56) ^+^(%)	10.76 (7.46, 17.37)	8.5 (6.48, 23.59)	-0.34	0.74
B factor (mg/dL)	32.9 (26.6, 38.2)	40.05 (32.28, 42.75)	-2.21	**0.03**
Immunoglobulin G (g/L)	10.8 (7.34, 12.2)	9.59 (8.9, 12.25)	-0.31	0.76
Immunoglobulin M (g/L)	0.98 (0.68, 1.58)	0.89 (0.61, 1.02)	-1.29	0.2
Complement 4 (g/L)	0.18 (0.16, 0.27)	0.22 (0.2, 0.26)	-1.9	0.06
Kappa-light chain (mg/dL)	857 (581, 963.5)	787.5 (762.75, 934.5)	-0.29	0.77
Lambda-light chain (mg/dL)	475 (320.5, 567.5)	423.5 (370.75, 555.5)	-0.18	0.86

Boldface values (all <0.05) indicate statistically significant differences in the corresponding variables between autoimmune encephalitis and viral encephalitis groups.

### 3.3 Flow cytometry results

We detected several co-stimulatory molecules on T cells, B cells, and monocytes by fluorescence-activated cell sorting (FACS). The results demonstrated that the following three key indicators were significantly higher in the early AE group compared to the early VE group, with statistically significant differences between the two groups (P<0.05). The median and IQR of CD4^+^ICOS^+^ levels in the early AE group were 14.32 (12.53, 16.83), showing a pronounced increase compared to 12.4 (7.45, 16.65) in the early VE group. The median and IQR of CD4^+^CXCR5^+^ICOS^+^ levels in the early AE group were 10.08 (7.53, 13.97), significantly higher than the 8.08 (5.38, 11.21) observed in the early VE group (P < 0.05). The median and IQR of CD19^+^PD-L1^+^ levels in the early AE group were 21.53 (14.6, 36.33), significantly higher than those in the early VE group (12.5 (7.34, 20.38) (P < 0.05), further confirming the statistically significant difference between the two groups. There is no statistical significance (P>0.05) in the following indicators between the two groups: CD4^+^PD-1^+^(%), CD4^+^CXCR5^+^PD-1^+^(%), CD4^+^CXCR5^+^PD-1^+^ICOS^+^(%), CD19^+^(%), CD19^+^ICOSL^+^(%), CD14^+^(%), CD14^+^ICOSL^+^(%), and CD14^+^PD-L1^+^(%) (**Table 5**).

**Table 5:** Comparison of flow cytometry results between autoimmune encephalitis and viral encephalitis.

	AE(n=37)	VE(n=37)	Z value	P value
CD4^+^ICOS^+^ (%)	14.32 (12.53, 16.83)	12.4 (7.45, 16.65)	-2.116	**0.034**
CD4^+^PD-1^+^ (%)	13 (8.16, 17.73)	14.05 (10.35, 18.55)	-1.158	0.247
CD4^+^CXCR5^+^PD-1^+^ (%)	10.66 (7.95, 12.92)	10.23 (6.28, 13.96)	-0.3	0.764
CD4^+^CXCR5^+^ICOS^+^ (%)	10.08 (7.53, 13.97)	8.08 (5.38, 11.21)	-2.181	**0.029**
CD4^+^CXCR5^+^PD-1^+^ICOS^+^ (%)	3.97 (1.86, 4.58)	2.61 (1.76, 4.23)	-1.235	0.217
CD19^+^ (%)	14.1 (9.04, 19.83)	16.8 (11.38, 22.6)	-0.964	0.335
CD19^+^ICOSL^+^ (%)	16.78 (9.79, 21.75)	12.15 (6.85, 20.45)	-0.858	0.391
CD19^+^PD-L1^+^ (%)	21.53 (14.6, 36.33)	12.5 (7.34, 20.38)	-2.769	**0.006**
CD14^+^ (%)	78.4 (73.2, 87.65)	86.8 (71.28, 96.2)	-1.346	0.178
CD14^+^ICOSL^+^ (%)	15.95 (8.91, 21.25)	12.6 (7.04, 28.08)	-1.035	0.301
CD14^+^PD-L1^+^ (%)	29.48 (13.6, 51.23)	14.65 (8.52, 38.08)	-1.569	0.117

Values are presented as median (M), 25% Interquartile range (Q1), and 75% interquartile range (Q3). AE, autoimmune encephalitis; VE, viral encephalitis.

Boldface values (all <0.05) indicate statistically significant differences in the corresponding variables between autoimmune encephalitis and viral encephalitis groups.

### 3.4 ELISA results

We detected several soluble co-stimulatory molecules in both plasma and CSF using ELISAs. Statistical analysis revealed significantly elevated plasma sICOSL levels in patients with early AE compared to those with early VE, demonstrating a highly significant intergroup difference (P<0.05). The mean and standard deviation of plasma sICOSL in the early AE group were 276.82 ± 91.3 ng/mL, showing a clear upward trend compared to 237.36 ± 84.78 ng/mL in the early VE group. Quantitative analysis revealed no statistically significant differences in the expression levels of additional soluble co-stimulatory molecules, including ICOS, PD-1, PD-L1, and CD14, between the early AE group and VE group (P > 0.05) (**Table 6**).

**Table 6:** Comparison of ELISA results between autoimmune encephalitis and viral encephalitis.

	AE(n=37)	VE(n=37)	Z value	P-value
Plasma ICOS (ng/mL)	35.16 (30.71, 40.92)	33.94 (32.16, 37.88)	-0.272	0.786
Plasma ICOSL (ng/mL)	276.82 ± 91.3	237.36 ± 84.78	2.037	**0.045**
Plasma PD-1 (ng/mL)	0.187 (0.111, 0.236)	0.15 (0.11, 0.22	-0.717	0.473
Plasma PD-L1 (ng/mL)	0.728 (0.55, 1.074)	0.754 (0.57, 1.07)	-0.086	0.931
Plasma CD14 (ng/mL)	5975 (4888.2, 6487)	5341.9 (4207.55, 6538.2)	-1.688	0.091
CSF ICOS (ng/L)	10.11 (8.87, 10.86)	9.6 (8.88, 10.85)	-0.241	0.83
CSF ICOSL(ng/ml)	9.85 (7.03, 11.90)	8.73 (6.69,14.56)	-0.076	0.939
CSF PD-1(ng/ml)	0.0005 (0, 0.03)	0 (0,0.06)	-0.561	0.575
CSF PD-L1(ng/ml)	0.16 (0.12, 0.24)	0.214 (0.139, 0.396)	-1.881	0.06
CSF CD14(ng/ml)	364.65 (236.18, 680.58)	401.5 (242.4, 950.2)	-1.107	0.268

Values are presented as median (M), 25% InterQuartile Range (Q1), 75% InterQuartile Range (Q3) and Mean ± standard deviation. AE, autoimmune encephalitis; VE, viral encephalitis.

Boldface values (all <0.05) indicate statistically significant differences in the corresponding variables between autoimmune encephalitis and viral encephalitis groups.

### 3.5 Model construction and assessment

Following comprehensive descriptive statistics and comparative analyses of the experimental data, we subsequently implemented univariate logistic regression analysis to evaluate potential associations. The results demonstrated statistically significant differences (P<0.05) between early AE and VE across multiple factors. These included serum creatinine [odds ratio (OR) = 1.033, 95% CI: 1.001–1.066, P = 0.046], chloride (OR = 1.187, 95% CI: 1.029–1.369, P = 0.019), calcium levels (OR = 0.019, 95% CI: 0.000–0.743, P = 0.034), CD4^+^ICOS^+^ (OR = 0.865, 95% CI: 0.771–0.970, P = 0.013), and CD19^+^PD-L1^+^ (OR = 0.961, 95% CI: 0.929–0.994, P = 0.020). Additionally, significant differences were observed in plasma sICOSL (OR = 0.994, 95% CI: 0.989–1.000, P = 0.036), CSF glucose (OR = 0.451, 95% CI: 0.229–0.887, P = 0.021), and in symptoms such as fever (OR = 6.981, 95% CI: 2.534–19.235, P < 0.001), nausea and vomiting (OR = 4.317, 95% CI: 1.464–12.730, P = 0.008), upper respiratory symptoms (OR = 2.888, 95% CI: 1.014–8.227, P = 0.047), headache (OR = 3.322, 95% CI: 1.293–8.538, P = 0.013), mental and behavioral disorders (OR = 0.267, 95% CI: 0.097–0.729, P = 0.010), and cognitive impairment (OR = 0.291, 95% CI: 0.092–0.925, P = 0.036) (**Table 7**).

**Table 7:** Results of single-factor logistic regression.

	B	SE^a^	DF^b^	P-value	OR^c^	95% CI^d^	
						Lower limit	Upper Limit
Creatinine (umol/L)	0.032	0.016	1	0.046	1.033	1.001	1.066
Chloride (mmol/L)	0.171	0.073	1	0.019	1.187	1.029	1.369
Calcium (mmol/L)	-3.958	1.868	1	0.034	0.019	0.000	0.743
CSF glucose(mmol/L)	-0.797	0.345	1	0.021	0.451	0.229	0.887
CD4^+^ICOS^+^(%)	-0.145	0.058	1	0.013	0.865	0.771	0.970
CD19^+^PD-L1^+^(%)	-0.040	0.017	1	0.020	0.961	0.929	0.994
Fever	1.943	0.517	1	<0.001	6.981	2.534	19.235
Nausea and vomiting	1.463	0.552	1	0.008	4.317	1.464	12.730
Upper respiratory symptoms	1.061	0.534	1	0.047	2.888	1.014	8.227
Headache	1.201	0.482	1	0.013	3.322	1.293	8.538
Mental and behavioral disorders	-1.322	0.513	1	0.010	0.267	0.097	0.729
Cognitive impairment	-1.233	0.589	1	0.036	0.291	0.092	0.925
Plasma ICOS (ng/mL)	-0.006	0.003	1	0.036	0.994	0.989	1.000

SE, standard error; DF, degree of freedom; OR, odds ratio; 95% CI, 95% confidence interval; CSF, cerebrospinal fluid.

**Figure 2:**
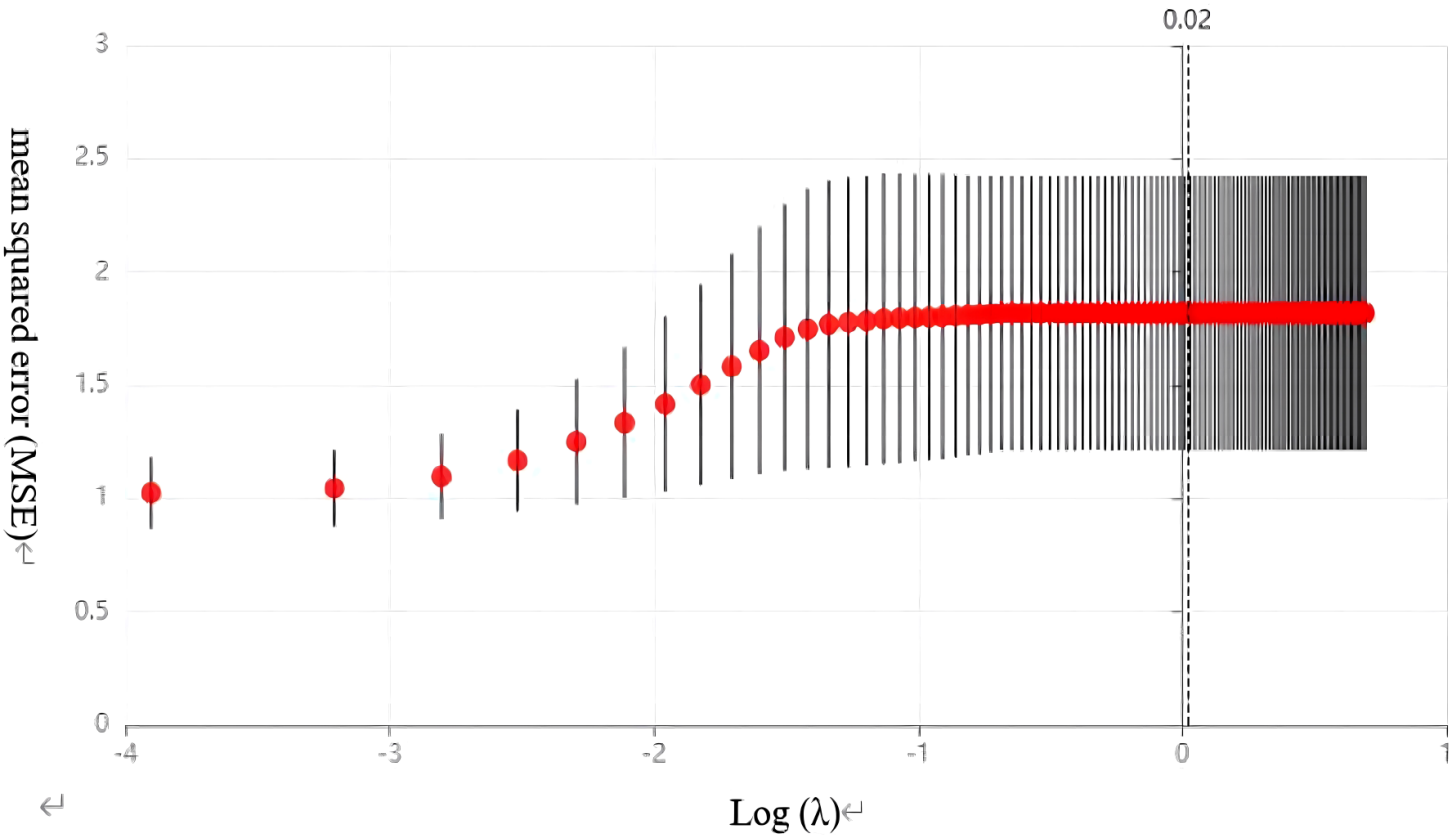
LASSO regression cross-validation plot. This figure shows the performance of the LASSO regression model under different regularization parameters (such as λ or alpha) using cross-validation to evaluate the model’s predictive accuracy. The horizontal axis represents the value of λ, and the vertical axis represents the model’s performance metric, namely, the mean squared error (MSE). In this figure, the optimal value of λ is 0.02.

**Figure 3:**
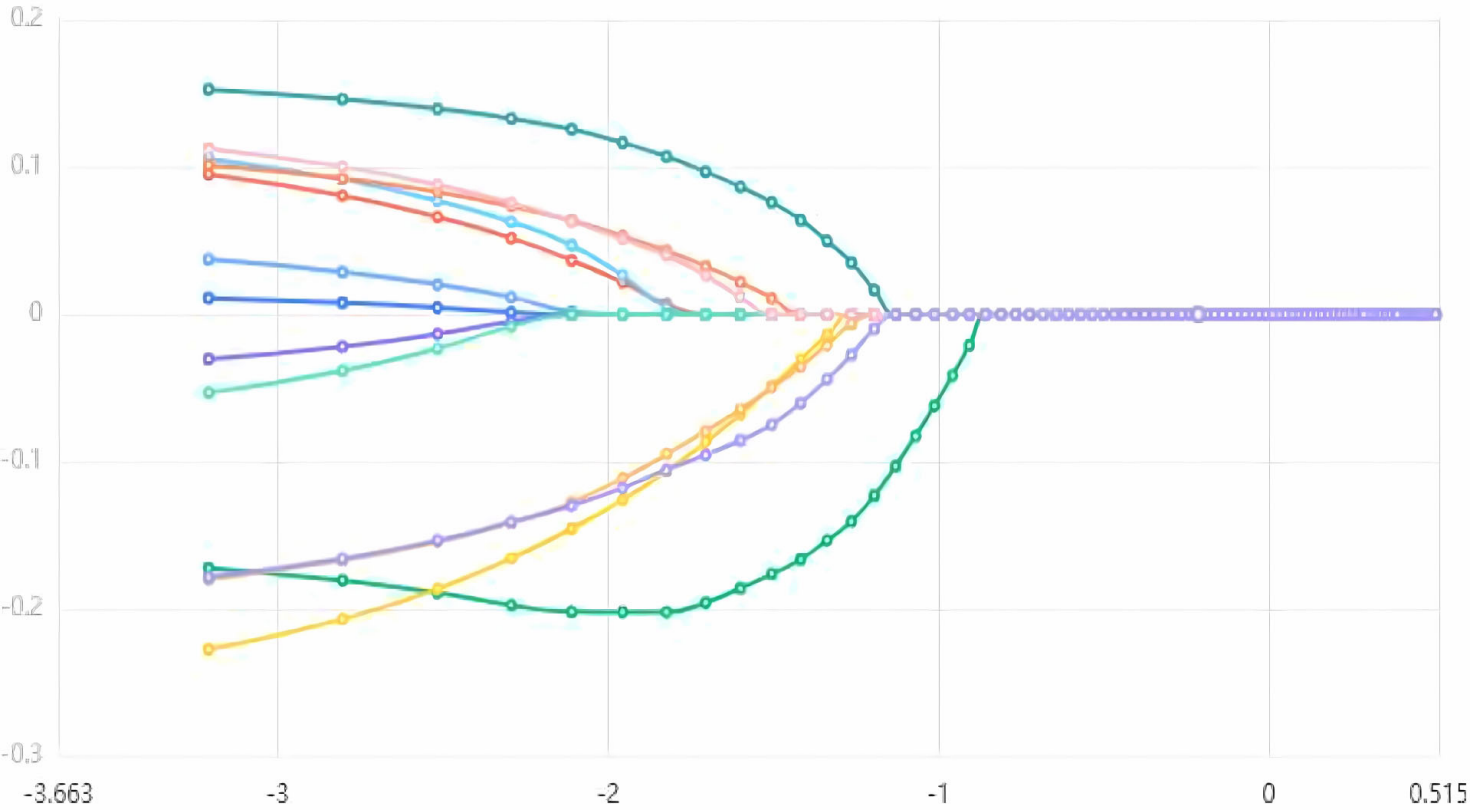
Graph of regression coefficients against γ value.

We performed a screening analysis of the aforementioned 13 variables using the LASSO regression method. After conducting cross-validation on the data and generating the corresponding plots, **Figure 2** illustrates the performance of the LASSO regression model under various regularization parameters (such as λ or alpha), thereby enabling the determination of the optimal λ value. In this analysis, the optimal λ value was identified as 0.02 (**Figure 2**). Additionally, **Figure 3** depicts a graph that was plotted to illustrate the relationship between λ and the corresponding variations in the model coefficients.

**Figure 4:**
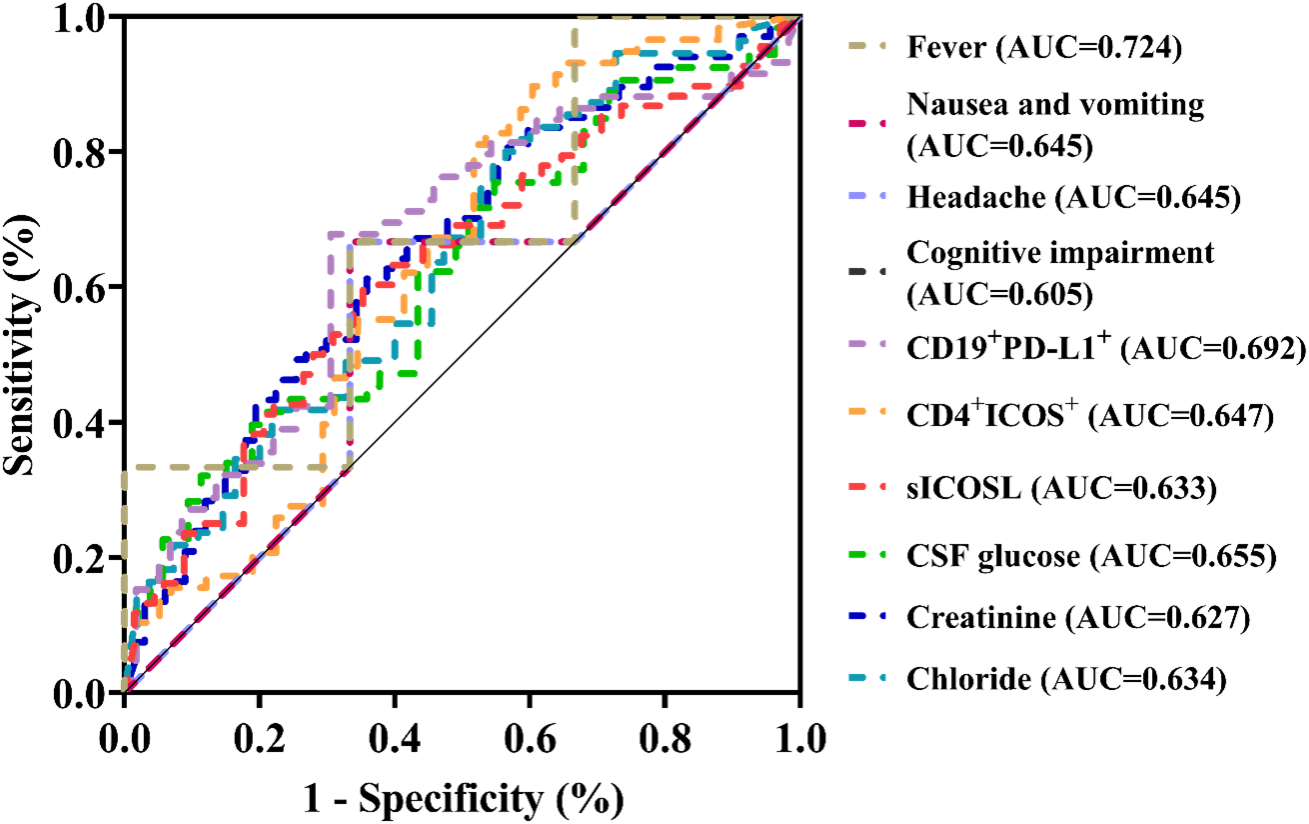
Various variables' receiver operating characteristic (ROC) curves. AUC (area under the curve) represents the area under the ROC curve and ranges from 0.5 to 1.0. The closer the value is to 1.0, the stronger the diagnostic capability of the variable.Furthermore, we constructed a standardized formula using the 10 selected variables: y (diagnosed with AE) = 4.508 + 0.016 × CSF glucose (mmol/L) - 0.108 × fever - 0.008 × creatinine (μmol/L) + 0.001 × plasma sICOSL (ng/mL) + 0.094 × Cognitive impairment - 0.071 × Nausea and vomiting + 0.021 × CD4^+^ICOS^+^ (%) - 0.128 × Headache + 0.005 × CD19^+^PD-L1^+^ (%) - 0.03 × Chloride (mmol/L). In the standardized formula, the threshold for y was determined based on the ROC curve of the model, selecting the point that maximizes the Youden index (sensitivity + specificity - 1), representing the optimal balance between sensitivity and specificity. For this model, the y threshold is 0.618. When y≥0.618, the patient is more likely to be diagnosed with AE.

Following LASSO regression analysis of the aforementioned 13 variables, we selected and retained 10 key variables: serum creatinine, chloride, CD4^+^ICOS^+^(%), CD19^+^PD-L1^+^(%), plasma sICOSL, CSF glucose levels, and clinical symptoms including fever, nausea and vomiting, headache, and cognitive impairment. A diagnostic model was constructed using the aforementioned 10 factors. Patients with elevated levels of CSF glucose and plasma sICOSL, increased percentages of peripheral blood CD4^+^ICOS^+^ and CD19^+^PD-L1^+^, and symptoms of cognitive impairment were more likely to be diagnosed with AE. Conversely, patients presenting with symptoms such as fever, nausea, vomiting, headache, and elevated levels of peripheral blood creatinine and chloride were more likely to be diagnosed with VE. Further analysis was performed on peripheral blood creatinine, chloride, CD4^+^ICOS^+^, CD19^+^PD-L1^+^, plasma sICOSL, and CSF glucose levels. ROC curves were generated to establish optimal cut-off values for differentiating AE from VE (**Figure 4**). The results indicated that patients with creatinine levels <60.75 μmol/L, chloride levels <106.25 mmol/L, CD4^+^ICOS^+^ ≥11.2%, CD19^+^PD-L1^+^ ≥12.35%, plasma sICOSL≥286.37 ng/mL, and CSF glucose levels ≥3.775 mmol/L were more likely to be diagnosed with AE. Considering the potential influence of factors such as age on the threshold values of the model variables, we explored the thresholds of variables including CD4^+^ICOS^+^, sICOSL, and creatinine across different age groups. This study considers the influence of age on the threshold values of the measurement data in the model. Based on age, patients with AE and VE were divided into three groups: 18–40 years as the young group, 41–60 years as the middle-aged group, and ≥61 years as the elderly group. The young group included 36 patients (17 AE, 19 VE), the middle-aged group included 23 patients (10 AE, 13 VE), and the elderly group included 16 patients (10 AE, 6 VE). The aforementioned study indicated that there was no significant difference in age distribution between the AE and VE groups during the early stage of the disease. We further calculated the discrimination thresholds for each quantitative parameter in the model across different age groups. The results showed that the discrimination thresholds for creatinine in the young, middle-aged, and elderly groups were 62.45 μmol/L, 54.55 μmol/L, and 44.25 μmol/L, respectively; for serum chloride, they were 106.8 mmol/L, 104.65 mmol/L, and 102.1 mmol/L, respectively; for the proportion of peripheral blood CD4^+^ICOS^+^, they were 12.05%, 14.1%, and 14.5%, respectively; for the proportion of peripheral blood CD19^+^PD-L1^+^, they were 12.55%, 11.85%, and 12.05%, respectively; for plasma sICOSL, they were 308.57 ng/mL, 186.70 ng/mL, and 294.45 ng/mL, respectively; and for cerebrospinal fluid glucose, they were 3.38 mmol/L, 3.42 mmol/L, and 3.64 mmol/L, respectively. The above findings suggest that the threshold values of the model variables may be influenced by age-related factors, which could be attributed to variations in physiological conditions across different age groups.

In the diagnostic model, variables such as cognitive impairment and headache demonstrated relatively low AUC values, suggesting only moderate diagnostic accuracy when considered independently. Although these variables are not strong standalone predictors, they still provide valuable supplementary information to the model. Our variable selection strategy emphasizes optimizing the overall performance of the model rather than prioritizing the performance of individual variables. While variables with lower AUC values serve as complementary factors rather than primary diagnostic indicators, their inclusion, when combined with other model variables, enhances the model’s overall predictive accuracy and strengthens its ability to facilitate early detection.

**Figure 5:**
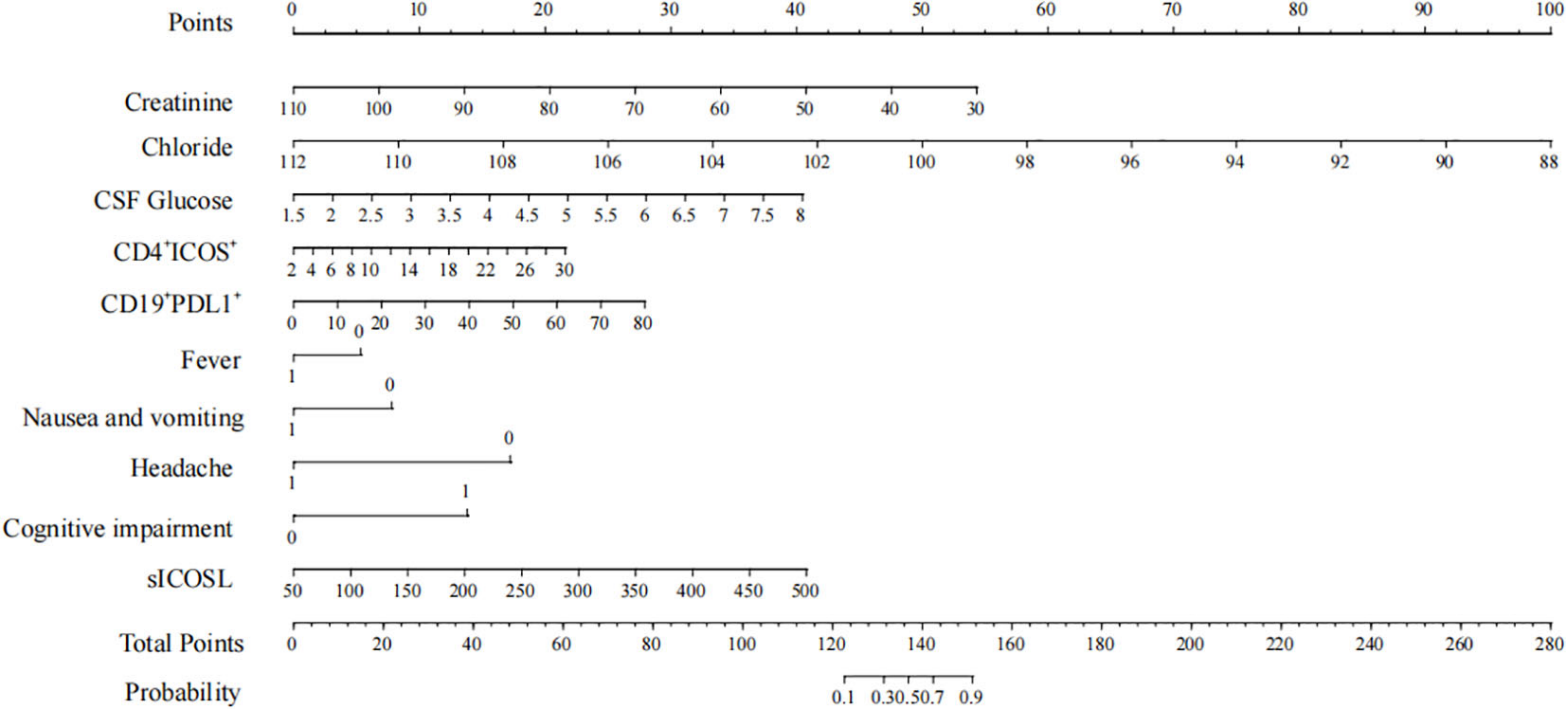
Nomogram points.

The model was subsequently visualized using a nomogram (**Figure 5**). The nomogram provides a practical tool for applying this model. By summing the scores corresponding to individual patient variables, the total score can be used to estimate the probability of an AE diagnosis. For example, consider a patient with the following characteristics: cognitive impairment (15 points), CD4^+^ICOS^+^ of 30% (22.5 points), CD19^+^PD-L1^+^ of 80% (27.5 points), plasma sICOSL of 500 ng/mL (40 points), and CSF glucose of 8 mmol/L (40 points). The total score for this patient is 145 points, corresponding to an 85% probability of an AE diagnosis.

**Figure 6:**
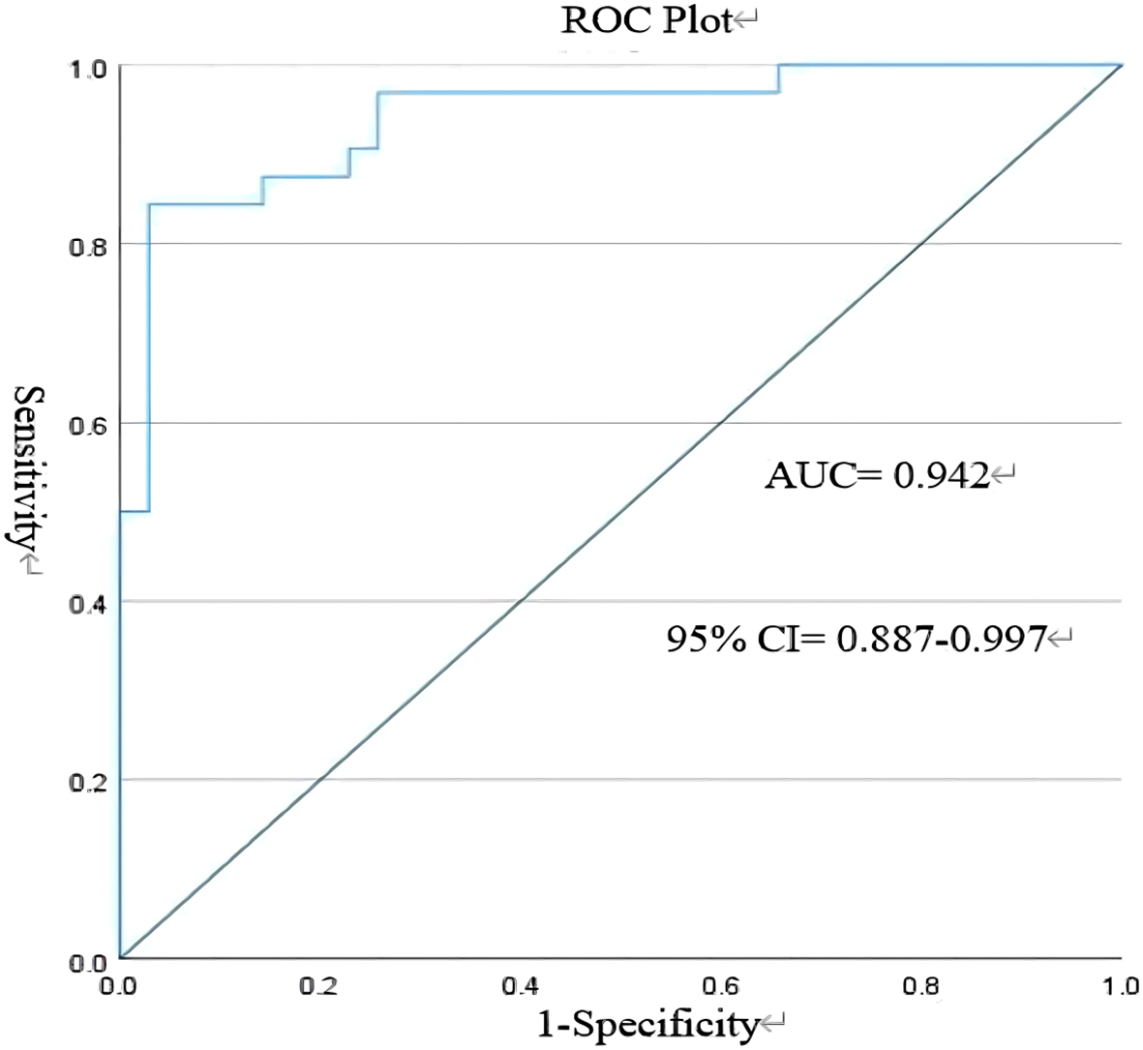
Receiver operating characteristic (ROC) curve. CSF, cerebrospinal fluid.

The model’s performance in discriminating early AE from VE was evaluated using an ROC curve (**Figure 6**). The AUC was calculated to be 0.942, with a 95% CI of 0.887 to 0.997. At the optimal threshold of 0.618, the sensitivity was 0.844, the specificity was 0.971, and the Youden Index was 0.815, demonstrating the model’s strong diagnostic utility.

## 4 Discussion

In this retrospective observational study, we investigated the differences in demographic and clinical characteristics, laboratory test results, peripheral blood flow cytometry findings, and ELISA results between early-stage AE and VE. Furthermore, we developed an early differential diagnosis model based on serum creatinine, chloride, peripheral blood CD4^+^ICOS^+^, CD19^+^PD-L1^+^, plasma sICOSL, CSF glucose, fever, nausea and vomiting, headache, and cognitive impairment. This model provides valuable guidance for clinicians in early-stage disease differentiation, and a nomogram was created to facilitate the accurate calculation of individual patients’ predicted probabilities.

A total of 19 indicators demonstrated statistically significant differences. The early VE group exhibited higher significance in fever, nausea and vomiting, upper respiratory symptoms, headache, serum chloride, creatinine, cerebrospinal fluid pressure, and white blood cell count. In contrast, abnormal psychological behaviors, cognitive impairments, serum LDH, serum calcium, CSF glucose, serum oligoclonal bands (OCBs), CD4^+^ICOS^+^, CD4^+^CXCR5^+^ICOS^+^, CD19^+^PD-L1^+^, and plasma sICOSL were more prominent in the early AE group. Kong et al. (23) previously developed a diagnostic prediction model distinguishing autoimmune limbic encephalitis (ALE) from viral encephalitis, incorporating eight indicators, namely age of onset, speed of onset, fever, headache, nausea and vomiting, neurological or memory disorders, status epilepticus, and CSF white blood cell count. Compared to their model, our study additionally incorporated peripheral blood flow cytometry analysis, focusing on the expression level of co-stimulatory molecules.

The immune system plays a critical role in both AE and VE. In most patients with AE, B cells produce autoantibodies targeting neuronal cell surface antigens, triggering neuronal dysfunction through various mechanisms and driving disease progression (24). In viral infections, the innate immune system is activated early, followed by the adaptive immune response, where immune cells and antibodies combat the viral pathogen (25). Anti-NMDAR encephalitis can occur as a complication of VE following an HSV infection (26).

Co-stimulatory molecules, which serve as indicators of immune status, are categorized into positive (e.g., OX40/OX40L, ICOS/ICOSL) and negative (e.g., PD-1/PD-L1, B7H3/B7H4) molecules. These molecules regulate T-cell activation and immune responses (27). CD4^+^ T cells promote inflammation through cytokine release and enhance B cell activation and antibody production (28). Membrane-bound ICOS/ICOSL, expressed on T cells and activated natural killer (NK) cells (29), plays a pivotal role in T follicular helper (Tfh) cell differentiation, germinal center formation, and antibody production (27). Conversely, PD-1/PD-L1 inhibit T cell activation, differentiation, and proliferation, regulating immune tolerance and responses to infections (30). In this study, membrane-bound ICOS (mICOS) on CD4^+^ T cells, membrane-bound PD-L1 (mPD-L1) on CD19^+^ B cells, and plasma sICOSL were significantly elevated in early AE compared to early VE, which is consistent with prior findings. In the early stages of neuromyelitis optica spectrum disorders (NMOSDs), membrane-bound ICOS/ICOSL, membrane-bound PD-1/PD-L1, soluble sICOSL, and soluble PD-1/PD-L1 also exhibited significant upregulation. However, peripheral blood-derived soluble sPD-1/sPD-L1 hinders the membrane PD-1/PD-L1 pathway, thereby augmenting T cell activity (31). Therefore, although the levels of membrane PD-1/PD-L1 are elevated, the positive signal still predominates and promotes immunity.

This study demonstrated that early AE is predominantly characterized by cognitive impairment, whereas early VE primarily manifests with fever, nausea and vomiting, and headache. Consistent with previous research, VE frequently presents with fever, headache, and vomiting as initial symptoms, distinguishing it from AE. Consequently, in patients presenting with these symptoms and suspected of encephalitis, viral encephalitis should be prioritized as a primary diagnostic consideration (32). Sakharova et al. identified cognitive impairment as a prominent feature in the clinical presentation of autoimmune encephalitis (33). Furthermore, in contrast to patients with VE, those with AE exhibit a higher prevalence of psychiatric and behavioral abnormalities, along with cognitive impairments (34). These findings are consistent with the results of the present study. In the early disease identification model developed by Granillo et al., four variables were incorporated: acute or subacute onset, symptoms associated with mental or memory impairment, a Charlson comorbidity index (CCI) score of less than 2 points, and a weaker inflammatory response in the cerebrospinal fluid (34). Notably, one of the variables in this model, psychological and memory-related symptoms, aligns closely with the symptoms examined in our study. One of the key components of the CCI involves scoring based on a patient’s comprehensive medical history. However, this study did not analyze the patient’s complete medical histories or positive physical findings, underscoring the need for further refinement and enhancement in future research.

Serum creatinine and chloride were incorporated into our differential diagnosis model. Additionally, there are significant differences in lactate dehydrogenase and serum calcium levels between early AE and VE. The levels of chloride and creatinine were found to be higher in VE, whereas lactate dehydrogenase and calcium levels were higher in AE. Creatinine, a byproduct of creatine metabolism, may rise in early VE due to increased energy demands (35). The serum creatinine levels were found to be significantly elevated in early untreated VE patients compared to those with early AE. VE patients who frequently use acyclovir may encounter acute kidney injury, thereby causing an increase in creatinine levels (36, 37). However, to date, there is a dearth of research investigating the correlation between serum creatinine levels and untreated viral encephalitis during its early stages. Creatinine levels may rise in early VE, possibly due to its involvement in the energy supply process. Studies have demonstrated an association between serum creatinine and a multitude of neurodegenerative disorders, with elevated levels of serum creatinine exhibiting a neuroprotective effect. The presence of elevated creatinine levels in female patients with anti-NMDAR encephalitis is inversely associated with the occurrence of psychiatric symptoms (38).

In this study, electrolyte imbalances were observed in both AE and VE, as evidenced by significantly elevated chloride levels in the early VE group compared to the early AE group and significantly higher calcium levels in the early AE group compared to the early VE group. Previous studies have primarily focused on blood sodium levels, revealing that hyponatremia is a common feature of anti-LGI1 encephalitis. This condition is linked to disorders of antidiuretic hormone secretion, resulting from hypothalamic involvement due to antibody-antigen reactions, as well as dysfunction of the medial temporal lobe (MTL) and basal ganglia (BG), which contribute to sympathetic dysfunction (39). In contrast, VE can present with either hypernatremia or hyponatremia, along with hypocalcemia, which are associated with the pathophysiological mechanisms of viral encephalitis (40). Notably, this study did not identify a significant difference in serum sodium levels between the two groups; however, a marked difference was observed in serum chloride levels. To date, no research has explored the relationship between serum chloride levels and encephalitis. The observed variation in serum chloride levels may be related to the distinct pathological processes underlying AE and VE.

Lactate dehydrogenase is found in the nerve cells of the brain, and it is released into the bloodstream when nerve cells are damaged. Central nervous system diseases such as cerebral infarction and hypoxic-ischemic encephalopathy are associated with elevated levels of serum lactate dehydrogenase (41, 42). Research indicates that patients with acute viral encephalitis demonstrate significantly higher LDH levels in their blood compared to healthy individuals (43). The serum LDH level was found to be higher in early AE patients compared to early VE patients in this study. However, there is a scarcity of studies that have directly compared the serum LDH levels between these two groups, necessitating further research in this area.

The CSF pressure and white blood cell count were significantly higher in early VE compared to early AE, whereas the CSF glucose content was notably elevated in early AE relative to early VE. A previous study revealed that both the VE and AE groups exhibited elevated levels of leukocytes and protein in the CSF, with the VE group demonstrating a more pronounced increase (32). In contrast to the VE group, the AE group showed no significant inflammatory response in the CSF, characterized by a white blood cell count of less than 50 per microliter and a protein content below 50 milligrams per liter (34). These findings are consistent with the results of the present study. CSF glucose levels are typically reduced in infectious diseases affecting the central nervous system. In this study, we observed that the CSF glucose content in early VE was significantly lower compared to early AE. To date, no studies have directly compared the cerebrospinal fluid glucose levels between AE and VE, necessitating further validation with larger sample sizes in future research. Additionally, investigations into the underlying reasons and mechanisms for the observed differences in cerebrospinal fluid glucose content between AE and VE are warranted.

It is acknowledged that this study has certain limitations. (1) Head MRI and EEG, which are valuable diagnostic tools for distinguishing between AE and VE, were not included in the examination protocol. In the current study, the exclusion of MRI and EEG data was primarily due to the following reasons. First, our cohort did not consistently include MRI and EEG data for all participants, which limited our ability to incorporate these variables into the model. Second, we focused on constructing a preliminary model by leveraging more accessible clinical and demographic data to identify and differentiate between AE and VE at an early stage. We fully acknowledge that the absence of these key diagnostic dimensions is a limitation of our current work. To address this, in subsequent research, we plan to incorporate MRI, EEG, and other neuroimaging data into our model to conduct a more comprehensive differential diagnosis between AE and VE. (2) We did not include cytokines in our analysis to differentiate AE from VE, as these markers are not routinely tested and are more expensive. (3) This study lacks external validation, making it impossible to evaluate the model’s potential for generalization. The primary objective of this study was to develop an early diagnostic model using the available data from our research cohort. Although the model demonstrates relatively strong diagnostic performance, it lacks external validation, which limits the assessment of its adaptability and generalizability. Additionally, autoimmune encephalitis is a rare disease, with an annual incidence of approximately one in ten thousand. Given the relatively short data collection period and the single-center design of this study, the sample size obtained is limited and insufficient for division into a training set and a validation set to perform internal cross-validation. To address these limitations, we plan to undertake the following steps in future research. First, we will actively seek collaborations with other institutions to access independent cohorts for external validation. Second, we intend to conduct a prospective study to further evaluate the reliability and generalizability of this model. Finally, we will explore the use of publicly available datasets to validate our model and enhance its credibility. (4) The sample size of this study is relatively small. A limited sample size and a single-center design may compromise the generalizability and robustness of the research findings. A small sample size can reduce the applicability of the results to broader populations and may lead to instability in the findings, potentially resulting in inconsistent outcomes if the study were replicated. Despite these limitations, we believe that our findings provide valuable preliminary insights into the early diagnosis of autoimmune encephalitis. In future work, we plan to collaborate with other institutions to collect multi-center data and expand the sample size. Additionally, we will extend the study duration by gathering data over a longer period to further increase the sample size and enhance the reliability of our conclusions. (5) Our study has a significant limitation: we did not collect detailed past medical history data from the patients. As a result, we were unable to perform a differential diagnosis analysis between patients with autoimmune encephalitis secondary to viral encephalitis and those with primary viral encephalitis. HSE-induced autoimmune encephalitis, clinically referred to as “double-peak encephalitis,” exhibits a distinct biphasic pattern. This condition is marked by initial clinical improvement following antiviral treatment during the acute phase of HSE, followed by the recurrence of neurological symptoms within several weeks to months (44). This temporal pattern is closely linked to the development of neuronal antibodies. Current evidence indicates that autoimmune encephalitis typically emerges within 2 months after HSE treatment. However, a critical limitation of this study is the lack of comprehensive historical medical data, which prevents us from conducting a differential analysis between double-peak encephalitis and viral encephalitis. In future research, we plan to collect detailed past medical histories of enrolled patients and categorize those with a history of viral encephalitis into a separate subgroup for more in-depth comparative analysis. This approach will enable a more comprehensive understanding of disease progression and help establish clearer diagnostic criteria.

Though there were some limitations, several advantages were evident in the study. First, the predictive factors incorporated in our model are readily accessible in clinical practice, thereby enhancing its practicality. ICOS/ICOSL and PD-1/PD-L1 can be detected via flow cytometry and ELISA, which represent short-term, convenient, and economical detection approaches. Other clinical manifestations and laboratory results were obtained as part of the routine admission examination. In addition, our model can provide a preliminary diagnosis of AE and VE, enabling us to determine whether to prioritize neuroantibody testing or perform mNGS testing for patients with limited financial resources.

## 5 Conclusion

In conclusion, utilizing clinical features, laboratory test results, flow cytometry data, and ELISA findings, we developed a diagnostic prediction model for the early differentiation of AE from VE. The model demonstrated strong discriminative performance, with high sensitivity and specificity. Furthermore, the variables included in the model are readily accessible, enabling clinicians to distinguish AE from VE at an early stage. This facilitates timely intervention, improves patient prognosis, and reduces the economic burden on patients.

## Data Availability

The datasets presented in this article are not readily available due to patient privacy considerations. Requests to access the datasets should be directed to Dr. Xue at qxue_sz@163.com.

## Supplementary material

Supplementary Table 1 Comparison of blood cell analysis between Autoimmune Encephalitis and Viral Encephalitis.

## Glossary

AE: Autoimmune encephalitis
VE: Viral encephalitis
CSF: Cerebrospinal fluid
NMDAR: N-methyl-D-aspartate receptor
MRI: Magnetic resonance imaging
EEG: Electroencephalogram
HSE: Herpes simplex encephalitis
mNGS: Metagenomic next-generation sequencing
NLR: Neutrophil-to-lymphocyte ratio
LGI1: Leucine-rich glioma-inactivated protein 1
GABABR: γ-aminobutyric acid B receptor
GAD: Glutamic acid decarboxylase
MOG: Myelin oligodendrocyte glycoprotein
DPPX: Dipeptidyl peptidase-like protein 6
TBA: Tissue-based assay
Tmax: Temperature maximum
TBil: The total bilirubin
DBil: Direct bilirubin
IBIL: Indirect bilirubin
ALT: Alanine aminotransferase
AST: Aspartate aminotransferase
GGT: Gamma-glutamyltransferase
TG: Triglycerides
LDL: Low-density lipoprotein
LDH: Lactate dehydrogenase
CK: Creatine kinase
α-HBDH: α-hydroxybutyric dehydrogenase
CRP: C-reactive protein
TC: Total cholesterol
ALP: Alkaline phosphatase
HDL: High-density lipoprotein
CSF MNC percentage: CSF mononuclear cell percentage
CSF PMN percentage: CSF polymorphonuclear cell percentage
HF-BF%: High fluorescence intensity cell percentage
CSF ADA: CSF adenosine deaminase
CSF LDH: CSF lactate dehydrogenase
CSF QIgG: CSF/serum IgG gradient
CSF IgG index: CSF immunoglobulin G index
CSF Q(A1b): CSF albumin ratio
CSF IgG: CSF Immunoglobulin G
CSF OCBs: CSF oligoclonal bands
SE: Standard error
DF: Degree of freedom
OR: Odds ratio
95% CI: 95% confidence interval
λ: Regularization parameters
MSE: Mean squared error
ROC curve: Receiver operating characteristic curve
AUC: Area under the curve
ALE: Autoimmune limbic encephalitis
HSV: Herpes simplex virus
NK cell: Natural killer cell
Tfh cell: T follicular helper cell
NMOSD: Neuromyelitis optica spectrum disorders
CCI: Charlson comorbidity index
MTL: Medial temporal lobe
BG: Basal ganglia
18F-FDG PET/CT: 18F-Fluorodeoxyglucose positron emission tomography/computed tomography
FACS: Fluorescence-activated cell sorting
IQR: Interquartile range
CD4-PC7: PE/Cyanine 7 anti-human CD4
ICOS-FITC: FITC anti-human/mouse/ral CD278 (ICOS)
CD19-FITC: FITC anti-human CD19
PD1-PC5.5: PerCP/Cyanine 5.5
CXCR5-PE: PE-anti-human CD185 (CXCR5)
OX40-apc: APC anti-human CD134(OX40)
TIM3-apc-cy7: APC/Cyanine 7 anti-human CD366(Tim-3)
CD14-pc7: PE/Cyanine 7 anti-human CD14
CD16-apc-cy7: APC/Cyanine 7 anti-human CD16
ICOSL-apc: APC anti-human CD275 (B7H2, ICOSL)
OX40L-pe: PE anti-human CD252(OX40L)
PDL1-pc5.5: PerCP/Cyanine5.5 anti-human CD274(B7-H1, PD-L1)
ELISA: Enzyme-linked immunosorbent assay
ICOS: T-cell inducible costimulator
ICOSL: T-cell inducible costimulator ligand
PD-1: Programmed death-1
PD-L1: Programmed death-ligand 1
