# Supplementary material for: Development of a prediction model integrating PD-1 and ICOS for early differential diagnosis between autoimmune and viral encephalitis
Source: Front Immunol. 2025 Apr 25;16:1550963. doi: 10.3389/fimmu.2025.1550963 (PMC12061884; doi:10.3389/fimmu.2025.1550963)
Supplement: Supplementary Table 1 — Comparison of blood cell analysis between Autoimmune Encephalitis and Viral Encephalitis. [file Table1.docx]

Supplementary Table：

Comparison of blood cell analysis between Autoimmune Encephalitis and Viral Encephalitis

|  | AE（n=37） | VE（n=37） | t/Z | P value |
| --- | --- | --- | --- | --- |
| Monocyte counts (10^9/L) | 0.47(0.35,0.66) | 0.55(0.42,0.75) | -1.31 | 0.189 |
| Neutrophil counts (10^9/L) | 5.45(3.71,7.88) | 5.94(3.73,7.27) | -0.34 | 0.738 |
| Neutrophil percentage (%) | 69(57.95,80.15) | 72.8(64.05,80.5) | -1.06 | 0.289 |
| Hemoglobin content ‌(g/L) | 134(117,145) | 135(121,141) | -0.28 | 0.779 |
| platelet counts (10^9/L) | 219(173,270) | 241(211,283) | -1.29 | 0.196 |
| Red blood cell counts (10^9/L) | 4.45(3.95,4.67) | 4.32(4.02,4.75) | -0.02 | 0.983 |
| White blood cell counts (10^9/L) | 8.36±3.15 | 8.16±2.79 | 0.28 | 0.78 |
| Lymphocyte counts (10^9/L) | 1.57±0.61 | 1.51±0.6 | 0.41 | 0.68 |
| Lymphocyte percentage (%) | 22.35±10.8 | 20±8.58 | 1.04 | 0.3 |
| monocyte percentage (%) | 6.81±3.25 | 7.13±2.67 | -0.46 | 0.65 |
| NLR^a^ | 2.95(1.83,6.21) | 3.56(2.49.5.70) | -0.781 | 0.435 |

Note: Values are presented as mean ± Standard Deviation or median (M), 25% InterQuartile Range (Q1) and 75% InterQuartile Range (Q3). a.NLR= neutrophil to lymphocyte ratio, is a reliable and easy available marker of immune response to various infectious and non-infectious stimuli; b. AE= autoimmune encephalitis, VE= viral encephalitis.
